# Challenges and opportunities for spiritual care practice in hospices in a middle-income country

**DOI:** 10.1186/s12904-021-00756-9

**Published:** 2021-04-22

**Authors:** Ronita Mahilall, Leslie Swartz

**Affiliations:** 1 Department of Psychology, Stellenbosch University, Stellenbosch, South Africa; 2 Department of Psychology, Stellenbosch University, Private Bag X4, Cape Town, 7745 South Africa

**Keywords:** Spiritual care, Palliative care, South Africa, Diversity, Hospice

## Abstract

**Background:**

Spiritual care is a key component of palliative care, but it has been overlooked and understudied in low- and middle-income country contexts, especially in Africa. In this study we sought to establish what the current spiritual care practices are in hospice palliative care settings in South  Africa with a focused view on what spiritual care training is currently offered and what training needs still remain unmet.

**Methods:**

We explored spiritual care practices, and training needs, through a national quantitative online study of palliative care organisations in South Africa registered with the Hospice Palliative Care Association of South Africa. A survey was sent to representatives of all member organisations listed on the national database of Hospice Palliative Care Association of South Africa. Viable data from 41% (*n* = 40) member organisations were analysed through the use of simple statistics.

**Results:**

An expressed need (75%; *n* = 30) was recorded for the development of a national spiritual care curriculum. Although 48% (*n* = 20) of the member organisations were willing to participate in the development of a spiritual care curriculum, 37% (*n* = 14) could not participate, citing financial (*n* = 27), time (*n* = 31) and expertise constraints (*n* = 22). A set of hard and soft skills were suggested to suit the diverse South African context.

**Conclusions:**

Spiritual care was seen by participants as a key component of palliative care. International  curricula  in spiritual care, while useful, do not offer easy adaptation to the diversities of South Africa. A bespoke spiritual care curriculum was called for, for diverse South Africa.

**Supplementary Information:**

The online version contains supplementary material available at 10.1186/s12904-021-00756-9.

## Introduction

Spiritual care is recognised as an essential element of holistic palliative care [1–3]. Spiritual care workers in the Global North^1^ are generally professional social workers [4–6], chaplains [7–9] and professional nurses [10–12], who are accustomed to having formal learning and teaching curricula. There is growing evidence that training in spiritual care may be associated with improved patient care outcomes [13, 14]. There is a plethora of studies in the Global North that support the need to have spiritual care training programmes in place for hospice staff [14–16].

In Africa, however, though spiritual care has been deemed important in the Global South [17–20], despite the spiritual diversity on the continent and the limitations on amassing meaningful national health care data [21], studies and protocols for the development of palliative care have tended not to focus on spiritual care, but rather on issues such as pain management protocols, for which there are clear indicators [22–25].

In 2017, the National Department of Health launched the South African National Policy Framework and Strategy on Palliative Care 2017–2022 [26]. The NPFSPC, which, like so many documents in SA, is an aspirational document focussing on the four main pillars of palliative care services: medical care services, psychosocial care services, spiritual care services, and bereavement care services.

The current study is based in South Africa (SA), where the previous oppressive apartheid regime used Christian religious ideology to justify oppression [27]. Emerging from a draconian past characterised by racial segregation and apartheid, and although well into its 26th year of democracy, SA still experiences the injustices of resource allocation, unequal educational and sparse learning opportunities. SA is also very diverse culturally, linguistically, and spiritually [28], with spiritual care interventions practised from a range of perspectives [29].

Given the complex South African context, the question arises as to whether there is a need for further training and development of spiritual care practices in palliative care. As part of a larger study, in this study we were interested to know what current spiritual care practices exist within hospice palliative care settings in SA. We were also interested to know from the perspective of hospices themselves, what their spiritual care training needs are and whether they see a need to develop a national curriculum for spiritual care intervention. We address these questions in this article.

## Methods

We conducted a simple Excel-based quantitative online survey (with some qualitative questions) (see Additional file 1) to address key questions on current spiritual care practices, and their perceived spiritual care training needs. The following are examples of the kinds of questions posed:

- What phrase best describes the extent of spiritual care offered at your hospice? (a. full care; b. partial care; c. occasional care; d. not at all);
- How is spiritual care practiced at hospices? (e.g. Does your organization recognize spiritual care as an integral part of a palliative care service it offers: a. Yes; b. No; c. If No, please elaborate.)
- Spiritual care training needs (e.g. Has a need for spiritual care training been expressed by your organization: a. Yes; b. No; c. We do not know at this time).

**Fig. 1:**
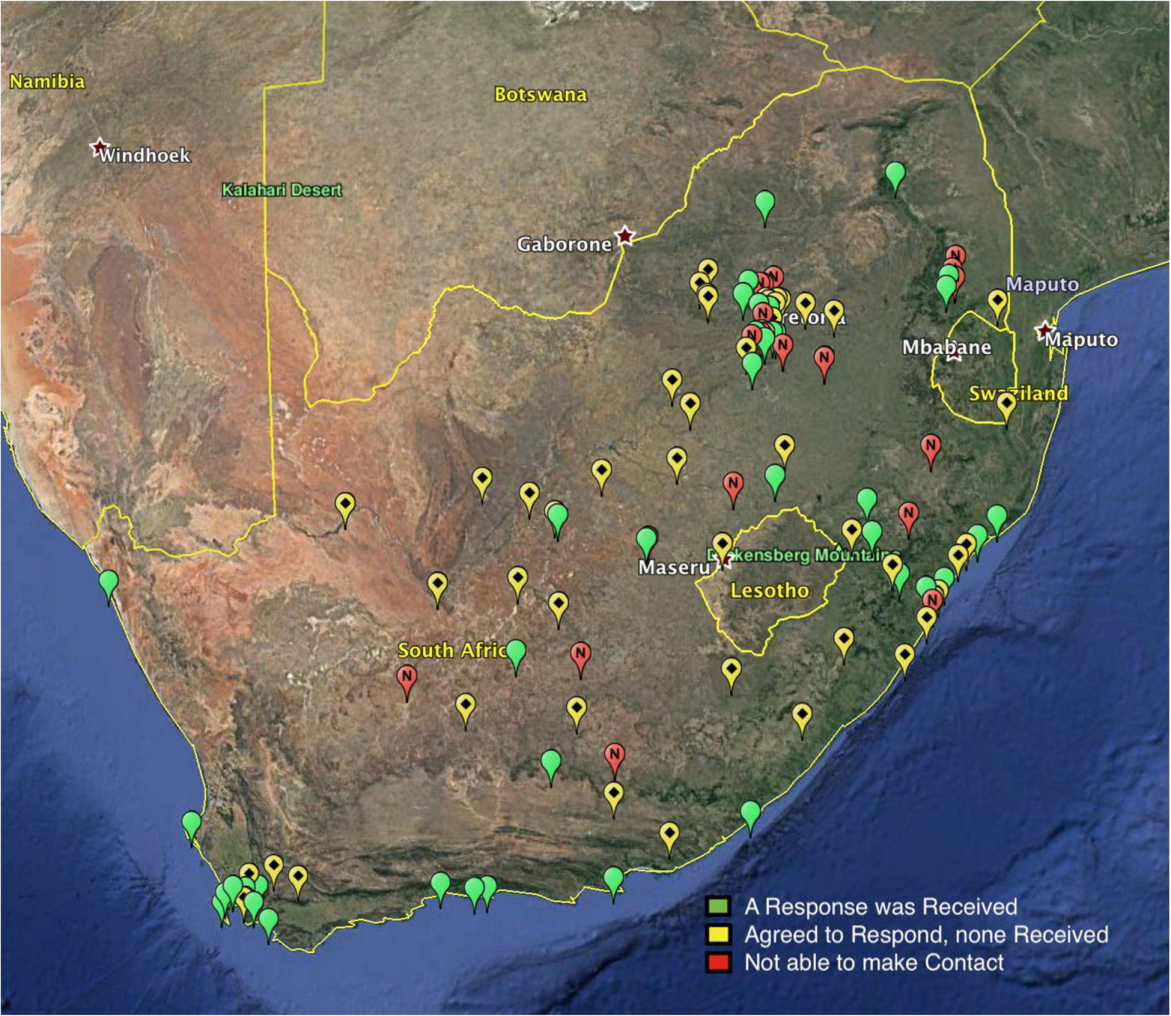
Mapping of hospices in SA

The comprehensive national database of Hospice Palliative Care Association (HPCA) of SA lists 104 member organisations. HPCA is a national association operating in all nine provinces in SA and 51 health districts. Some of its voluntary member organisations offer hospice palliative care, some home based care, and others both services. We use the term ‘member organisations’ rather than ‘hospices’ because all organisations offer palliative care, but not all call themselves hospices. Ethical approval was obtained from Stellenbosch University (10237) as well as HPCA (02/19). We telephoned the leader of each organisation, generally the Chief Executive Officer (CEO) or General Manager (GM), to position the study and to obtain their consent to be a part of this study. Further we engaged with them to establish who, within their organization, was best placed to participate in the survey. Ninety-six member organisations were contactable. We distributed the survey to all organisations, through their CEO or GM for them to assign to best placed staff to complete. Forty-three responses were received, 40 of which produced viable data. The completed surveys represent 41.6% (*n* = 40) of the contactable HPCA member organisations across SA. Written consent was obtained from participants of each hospice who participated in this study. We conducted a Geographic Information System (GIS) [30], mapping to observe the spatial pattern of responses relative to the location of all organisations (Fig. 1). The GIS mapping further shows the maldistribution of hospices in SA. Although SA is classified as a middle-income country, approximately 33% of the SA population live in rural areas, which by definition, are those areas that are without access to ordinary public services such as water and sanitation and are without a formal local authority, thereby significantly inhibiting rural development (World Bank Blog 2021/2022, Atlas). The survey results were then collated, analysed, and filtered for key issues and overarching themes.

## Results

As Fig. 1 shows, there does appear to be a clustering of responses received to the survey, and it appears further that the clusters of response tend to map on to major urban areas of SA, where there tends to be more health and other resources (NPFSPC, 2017–2022). This mapping also highlights the maldistribution of hospices in SA, not an unfamiliar reality given SA’s apartheid past. This needs to be borne in mind in the analysis of the data.

Table 1 provides an overview of palliative care service capacity in general in SA, as well as the outreach to patients. All nine provinces were represented. Member organisations reported a cumulative total of 7981 patients per month. When this amount was projected for the entire HPCA member organisations per province, the total number of patients cared for per month was 20,078.

**Table 1:** Provincial HPCA Member Organisations, Staff, and Patients’ Data

Province	Total Contactable HPCA Member Organisations in SA	Number of respondent HPCA Member Organisations	Total # Staff from Respondent HPCA Member Organisations	Patients per month	Total HPCA Member Organisation Projection of patients per month
Eastern Cape	6	3	77	690	1380
Free State	8	2	24	316	1264
Gauteng	18	8	372	1338	3010
Kwa-Zulu Natal	17	7	164	1385	3364
Limpopo	2	2	26	25	25
Mpumalanga	7	2	20	267	934
North West	8	2	17	615	2460
Northern Cape	14	3	41	864	4032
Western Cape	16		501	2481	3609
**Totals**	**96**	***n*** **= 40 (42)**	**1242**	**7981**	**20,078**

Some member organisations did not offer the full spectrum of palliative care services to their patients. One member organisation did not offer medical care services, and three member organisations did not offer psychosocial care services. Most member organisations described their spiritual care services to patients as ‘occasional’, with 58% (*n* = 23) making occasional external referrals outside their organisation, and 63% (*n* = 25) rendering occasional internal services. Where spiritual care services were generally available, they were rendered on a partial care basis, primarily through an in-house spiritual care team or this service was outsourced to spiritual care service providers located in the community such as traditional leaders, and faith-based organisations.

When asked to indicate the extent to which spiritual care services were integrated into palliative care at the respective member organisations, 30% (*n* = 12) of the member organisations replied, ‘fully integrated’, and 20% (*n* = 8) replied ‘partially integrated’.

Most (*n* = 27; 68%) member organisations indicated that they did not pay nor give incentives to their spiritual care workers, which suggests that spiritual care as largely a voluntary activity. Spiritual care workers form a diverse group, with isiZulu being the most widely spoken language amongst spiritual care workers, followed by English, Afrikaans and isiXhosa, and then a range of other African and non-African languages. Most spiritual care workers were reported to be Christian (*n* = 21), followed by Buddhist (*n* = 6), Muslim (*n* = 4), and Jewish (*n* = 2).

Only half of the 40 (*n* = 20) member organisations provided data on spiritual care workers’ levels of education and qualifications, and some of those that did highlighted that the data may not be accurate. Nine of the member organisations’ spiritual care workers had a school leaving certificate,^2^ followed by six of the member organisations’ spiritual care workers who had a tertiary undergraduate qualification, and five of the member organisations’ spiritual care workers who had a postgraduate qualification.

When member organisations were asked to provide details on the nature of spiritual care training the spiritual care workers had received, under two thirds (*n* = 25) of member organisations responded. Of those responses, member organisations indicated that only one in five of them (*n* = 8) had spiritual care workers that received advanced training in spiritual care. Roughly a third (*n* = 12) of member organisations had spiritual care workers who had received no formal training, and a fifth (*n* = 8) of member organisations had spiritual care workers who had received basic training in spiritual care. Considering that over a third of the member organisations did not provide data, the responses from the member organisations suggest significantly low levels of spiritual care training opportunities for their spiritual care workers.

Of the 40 member organisations, only four indicated that they had in place a spiritual care curriculum for training and skills development for spiritual care workers and for their other palliative care staff. Most (*n* = 33) of the member organisations had none. The majority of the member organisations reported that they were unable to provide, nor afford, any in-house spiritual care training and skills development for their spiritual care workers due to funding and expertise constraints (*n* = 33). By contrast, three quarters (*n* = 30) of the member organisations indicated a need for an established national baseline spiritual care curriculum, while only two did not, and two thirds (*n* = 26) of member organisations saw value in their spiritual care staff receiving spiritual care training. About half (*n* = 20) of the member organisations indicated willingness to participate in the development of a specialised curriculum for palliative care spiritual care training. Suggestions made by member organisations on how they could contribute to the development of such a curriculum included: through workshops (*n* = 25); through focus groups (*n* = 31); through brainstorming/think-tank sessions (*n* = 36).

Though half (*n* = 20) of the member organisations were willing to participate in the development of a spiritual care curriculum, most of them (*n* = 14) indicated that they would prefer not to participate right now. When asked if they would be able to fund sending their spiritual care workers for spiritual care training and skills development, just over half (*n* = 22) of member organisations indicated that they would, but not at the moment. Under a quarter (*n* = 9) stated that they would be willing to pay for such training for their spiritual care workers, and five organizations said they would not be able to afford to pay for such training. Some of the reasons member organisations offered for not participating in the development of a national spiritual care curriculum were: financial (*n* = 27) and time constraints (*n* = 31); and uncertainty as to what value they could bring to this process (*n* = 22).

Member organisations suggested the following sets of skills that ideally spiritual care worker needed to have in order to render quality spiritual care services in a diverse SA. Table 2 outlines those skills in order of prominence**.**

**Table 2:** Member organisations’ views on essential skills of spiritual care workers

Essential Skills of an Effective Spiritual Care Worker	
Hard Skills^a^	Soft Skills^b^
A sound understanding of palliative care, the context of palliative care in SA and its practice. (*n* = 32)	Strong counselling skills (*n* = 38)
Being able to communicate in at least 2 of the official languages in SA. (*n* = 29)	High levels of self-awareness, including insight into one’s own spirituality and tolerance for diversity (*n* = 31)
Knowledge and understanding of the integration of palliative care and spiritual issues in hospices and in SA. (*n* = 29)	High levels of acceptance, non-judgmental, and open mindedness. (*n* = 28)
Knowledge of patient rights and being able to clearly identify the needs of the patient. (*n* = 27)	The ability to develop a plan for personal, spiritual and professional growth, self-awareness and self-understanding. (*n* = 28)
Having knowledge of and insight into other cultures and religions and being sensitive when dealing with differences and diversity. (*n* = 27)	Maintains a well-articulated awareness of one’s own understanding of spirituality, religion, spiritual health and how to offer spiritual health care in a diverse clinical setting. (*n* = 28)
Knowledge of the medical sector, including professional hierarchies as well as detailed knowledge of the common terminal illnesses patients presents with and consequently being able to understand and respond appropriately to the impact those different illnesses have on the patient. (*n* = 21)	High levels of compassion, motivation, passion and enthusiasm for palliative care. (*n* = 23)

^a^Hard skills are teachable and measurable abilities, such as writing, reading, using computer programmes

^b^Soft skills are the traits that make a good team member, such as etiquette, communication and listening

## Discussion

Though member organisations were offering valued spiritual care services, to varying levels, in practice spiritual care services did not receive as much priority as medical or psychosocial services [29]. Funding, and expertise constraints seemed key to the lack of resourcing for spiritual care, and we discuss each in turn.

### Funding limitations

Local and international studies support the role of spiritual care in enhancing patient care outcomes [15, 31], yet funding constraints dictate that member organisations have to consciously pick and choose what they can afford to offer, often at the exclusion of spiritual care services. All the member organisations participating in this study are non-governmental organisations (NGOs), depending largely on donor funding to sustain their work. By virtue of being NGOs, member organisations provide a largely free service to the public. With ever-growing funding needs exacerbated by the COVID-19 virus, funding may well decrease rather than increase [32]. This is a clear threat to developing the spiritual care services organisations say they need, especially in a context of existing high palliative care and spiritual care demand and likely increase in demand given the COVID-19 pandemic.

### Expertise limitations

Member organisations clearly felt that they had limited expertise in spiritual care, and that this was a barrier in their participation in developing national spiritual care training curricula. This finding is in alignment with what Gijsberts et al. [33] found in a systematic review of recent studies on spiritual care in palliative care in Europe where spiritual care givers saw the provision of spiritual care as part of their role but felt less confident in their spiritual care competencies in that role. Gijsberts et al. [33] also found that there was an expressed desire by palliative care consultants for training to deal with spiritual issues.

The member organisations identified a set of generalist hard and soft skills that a spiritual care worker should ideally have in order to offer an effective spiritual care service, largely in keeping with those outlined elsewhere [34]. The hard skills slanted more towards the spiritual care workers having a good understanding of spirituality as a phenomenon; spirituality as a key component of a palliative care service; and a good understanding of the religious, cultural and linguistic diversities underpinning how the spiritual care service would be shaped and delivered in SA settings. Interestingly, a cohort of hospices in the Western Cape Province of SA mentioned similar skills-sets [29]. The soft skills, as suggested by the member organisations, consisted of more intangible and nuanced skills that are typically acquired through practice and experience, such as being present while paying attention to the spirituality of the patient and their hopes; being self-reflective; and bringing peace to the patients. These findings are mirrored by a Dutch study [35] where knowing one’s own spiritual background was also considered as part of the self-reflective journey of the spiritual care worker. The skills deficit expressed by member organisations, therefore, may relate not only to hard skills but also to anxiety about providing appropriate, non-discriminatory care in a diverse and highly politicised social environment such as SA. It may be that an important basis of any training would be to address, directly, the political and social context of palliative care, and spiritual care, a key issue for SA, but not addressed to any large degree in the spiritual care literature.

## Conclusion

SA is a young democracy, born out of considerable conflict but with aspirations to provide the best for all its diverse citizenry. This reality affects palliative care as it does all other aspects of SA society. Our data demonstrate the gap between actual practice and national policy, a not uncommon feature in SA [36]. Member organisations recognise the importance of spiritual care as a key component of palliative care but are acutely aware of the barriers that hinder such a consolidated approach. It is clear that the international models for the development of training and services in the field of spiritual care have much to offer the development of the field conceptually in SA, but that the local situation of a divided and diverse society, with considerable resource constraints, will necessitate a local, contextually relevant approach. In a number of fields of care in Africa, and in SA in particular, the judicious use of international guidelines tempered with local realities have been attempted [22–25]. We are not yet sure what an ideal model of spiritual care will look like practically in the SA context. In keeping with our informants in this study, however, we believe that this dimension of care is important and should be documented with prominence, and not hidden in palliative care frameworks for Africa.

## Supplementary Information

**Additional file 1.** Sub-study 1. Online survey of all HPCA member organisations in SA

## Data Availability

The datasets used and/or analysed during the current study are available from the corresponding author on reasonable request.

## Abbreviations

GIS: Geographic Information System
HPCA: Hospice Palliative Care Association of South Africa
SA: South Africa

## Footnotes

1 There are many debates about how to classify countries. The ‘Global North’ generally corresponds to countries categorised by the World Bank (World Bank Blog 2021/2022 Atlas) as high-income countries (HICs) – so, for example, Australia, which is a high-income country is commonly viewed as part of the Global North, despite its being in the southern hemisphere. Countries in the ‘Global South’ generally correspond to the World Bank low and middle-income countries (LMICs) (World Bank Blog 2021/2022 Atlas). South Africa is a middle-income country. The distinction here is based largely on financial resources, though of course other factors are associated with relative wealth.

2 A school leaving qualification is an academic qualification awarded for the completion of high school. Depending on the country or region, it may alternatively be known as a high school diploma, senior secondary leaving certificate, high school general certificate or school certificate. The National Senior Certificate or NSC is a high school diploma and is the school-leaving certificate in South Africa. This certificate is commonly known as the matriculation (matric) certificate, as grade 12 is the matriculation grade (http://ncdoe.ncpg.gov.za/ (accessed 30 March 2021)).
